# Impairment of affective and cognitive empathy in high functioning autism is mediated by alterations in emotional reactivity

**DOI:** 10.1038/s41598-024-71825-1

**Published:** 2024-09-17

**Authors:** Ann-Christin S. Kimmig, Lina Burger, Marina Schall, Birgit Derntl, Dirk Wildgruber

**Affiliations:** 1 Department of Psychiatry and Psychotherapy, University of Tübingen, Calwerstr. 14, 72076 Tübingen, Germany; 2 German Center for Mental Health (DZPG), Partner Site Tübingen, Tübingen, Germany; 3 Department of Psychology, University of Bamberg, Bamberg, Germany; 4 LEAD Graduate School & Research Network, University of Tübingen, Tübingen, Germany

**Keywords:** High-functioning autism, Autism spectrum disorders, Cognitive empathy, Affective empathy, Emotional reactivity, Textual empathy test, Autism spectrum disorders, Human behaviour

## Abstract

Empathy impairments are considered a key aspect of autism-spectrum disorders (ASD). Previous research consistently shows reduced cognitive empathy, but findings on affective empathy vary, possibly due to experimental design variations (e.g., stimulus modality, social distance) and individual psychological factors (e.g., perceptual abilities, emotional reactivity). This study aims to clarify deficits in affective and cognitive empathy in ASD by addressing these contributing factors. Empathy was examined in 34 autistic individuals and 33 typically developed controls (TDCs) through the Textual Empathy Test (TET). The TET was developed to assess emotional responses when imagining oneself (emotional reactivity) as compared to a target person (friend, stranger) in emotional situations presented via short verbal descriptions. Participants rated emotional states of the target person (cognitive empathy) as well as their own emotional responses when imagining the target person in that situation (affective empathy). Ratings were interpreted relative to normative mean values through standardized regression coefficients. Results showed that high-functioning autism was associated with lower cognitive and affective empathy irrespective of social distance as well as with decreased emotional reactivity compared to controls. Moreover, emotional reactivity mediated the impact of ASD on both empathic components. In summary, altered emotional reactivity may underlie impaired empathy in autistic individuals.

## Introduction

Major hallmark characteristics of autism spectrum disorder (ASD) include deficits in empathy as well as social interactions^1^. Empathy is crucial for social functioning and behaviors^2^, as it aids the understanding of another person’s affective state and consequently facilitates the appropriate response. Most commonly two components of empathy are proposed: the cognitive and the affective empathy (e.g.,^3–5^). While cognitive empathy refers to perspective-taking and understanding the emotional states of others, affective empathy denotes the conscious resonation of another’s feelings (i.e., experiencing congruent emotional states). Typically, cognitive and affective empathy are regarded as largely independent but highly intertwined processes^6^. Furthermore, empathy is at least partly based on or influenced by processes related to attention and emotion-recognition^3–5^.

Studies, including a recent meta-analysis^7^, indicate that autistic people tend to show decreased abilities in cognitive and affective empathy compared to individuals without autism (e.g.,^8–14^), which may contribute to difficulties in social interactions. While some studies suggest a more consistent and larger impairment of cognitive empathy (e.g.,^15–21^), findings regarding affective empathy are mixed. According to Bird and Viding^4^ ASD is not directly associated with impairments of sharing the emotions of others. Instead, deficits in affective empathy are primarily mediated by impairments in the perception of nonverbal emotional cues and reduced attention to socially relevant stimuli, among other factors.

Moreover, the own tendency to experience emotions (i.e., emotional reactivity) could affect the intensity of emotions attributed to and/or felt for others (i.e., cognitive and affective empathy, respectively^22–24^). Several studies suggest that (physiological) emotional reactivity is altered in ASD^25,26^. Depending on the context and interindividual variance, emotional reactivity may be partly increased (e.g.,^27,28^) or decreased (e.g.,^29–33^) compared to typically developed controls (TDCs).

Lastly, the occurrence of affective empathy deficits in autistic individuals may also depend on the social distance of the target person, as suggested by studies using self-report^15^ and pupillary measures^34^. Both studies indicate that affective empathy may be primarily impaired for distant but not necessarily close target persons. Taken together, factors such as impaired recognition of emotional faces and voices (see for meta-analysis:^35^), altered emotional reactivity in autistic people^25^ and the social distance of the target person may contribute to the greater heterogeneity in findings concerning affective compared to cognitive empathy.

The aim of the present study was to gain a better understanding of empathy deficits in high functioning autistic individuals by controlling for or taking mediating and moderating factors into account such as emotion recognition skills, deviant emotional reactivity and social distance to the person to be empathized with. To this end, we used the Textual Empathy Test (TET, originally “Tübinger Empathy Test” cf.^24,36^), which was adapted for the application in mixed-sex samples across a broader age range (see the methods section and supplementary information for information on task adaptation). In the TET, textual descriptions of emotional situations are presented to measure cognitive and affective empathic responses toward target persons with varying levels of social distance (i.e., close and distant person perspective) as well as one’s own emotional reactivity (i.e., self-perspective). The use of textual stimuli in the TET mitigates confounding effects of emotion recognition skills, e.g., in terms of facial expressions, gestures, and speech prosody. The emotional situations in the TET are balanced for eliciting positive (i.e., joy, hope, sexual arousal, gratefulness, pride) and negative emotions (i.e., shame, anger, disgust, sadness, fear) to avoid a bias towards negative emotions, for which previous empathy tasks have been criticized (e.g.,^37,38^).

Regarding empathic abilities, we were firstly interested whether the extent of empathic deficit varies for cognitive and affective empathy for autistic people. Following previous literature, we predicted impaired cognitive and affective empathy in autistic individuals compared to typically developed controls (e.g.,^8–14^). We additionally tested whether differences in the social distance to the target person (friend/family, stranger) would affect the extent of empathic responses of ASD and TDC individuals. Cognitive and affective empathic responses were operationalized using standardized regression coefficients (betas). Separate regression analyses were performed at the participant level, using normative mean ratings from an independent sample of typically developed individuals (n = 82) as predictors for the participants’ valence ratings, which indicated the emotions the target person would experience in the same situation (representing cognitive empathy) and the emotional valence felt when imagining the target person in this situation (representing affective empathy, see method section for more detailed information). This approach was chosen to create a more sensitive and standardized measure of how well the expected emotional reaction of a target individual aligns with the predicted emotional response of the target person (cognitive empathy) as well as the own emotional response (affective empathy) to another person’s experience.

Secondly, due to the possible link between emotional reactivity and empathic abilities^22–24^, we accounted for emotional reactivity in our study as potential mediating factor of empathy. We predicted from previous literature that autistic individuals will show reduced (or deviant) emotional reactivity when imagining emotional situations compared to TD controls^25,26^. Similar to empathic responses, we conducted within-individual regressions, correlating normative valence ratings with participants’ self-ratings, and utilized standardized regression coefficients to operationalize emotional reactivity.

Finally, we expected that deviant emotional reactivity in ASD could explain cognitive and/or affective empathy deficits associated with ASD. To test this prediction, we conducted additional analyses with emotional reactivity as potential mediator of empathy.

## Results

All following analyses were conducted on 34 high functioning autistic individuals and 33 typically developed controls, who were matched with regards to age, sex, education and IQ (see Table 1). Explorative analyses revealed no sex-related differences in standardized regression coefficients for empathic and emotional reactivity, therefore it was not included as a factor in the following analyses.

**Table 1:** Comparison of demographic and neuropsychological characteristics between ASD and TDC group (mean and standard deviation if not otherwise specified).

	ASD	TDC	p-value
N	34	33	
Age (years)	40.5 (12.1)	38.9 (10.8)	0.59
Sex (male/female)	25/9	24/9	0.94
Academic degree (no/yes)	21/12*	21/12	1.00
Verbal IQ (raw scores)	32.7 (3.5)	32.5 (2.6)	0.31
AQ (ASD traits)	38.7 (6.0)	15.0 (6.1)	< .001

*Missing data (n = 1).

*ASD* autism spectrum disorder, *TDC* typically developed controls, *IQ* intelligence quotient, *AQ* autism quotient.

### Cognitive and affective empathy in autistic individuals and typically developed controls

A 2(group: ASD, TDC) × 2(empathy component: cognitive, affective) × 2(social distance: friend, stranger) mixed ANOVA using standardized regression coefficients (betas) for empathic responses as the dependent variable was run to investigate the first hypothesis and explorative questions concerning ASD and empathy. Cognitive and affective empathic response betas were determined through regression analyses of each participant’s respective valence ratings (dependent variables) with normative data from an independent sample of TD individuals (predictor).

**Fig. 1:**
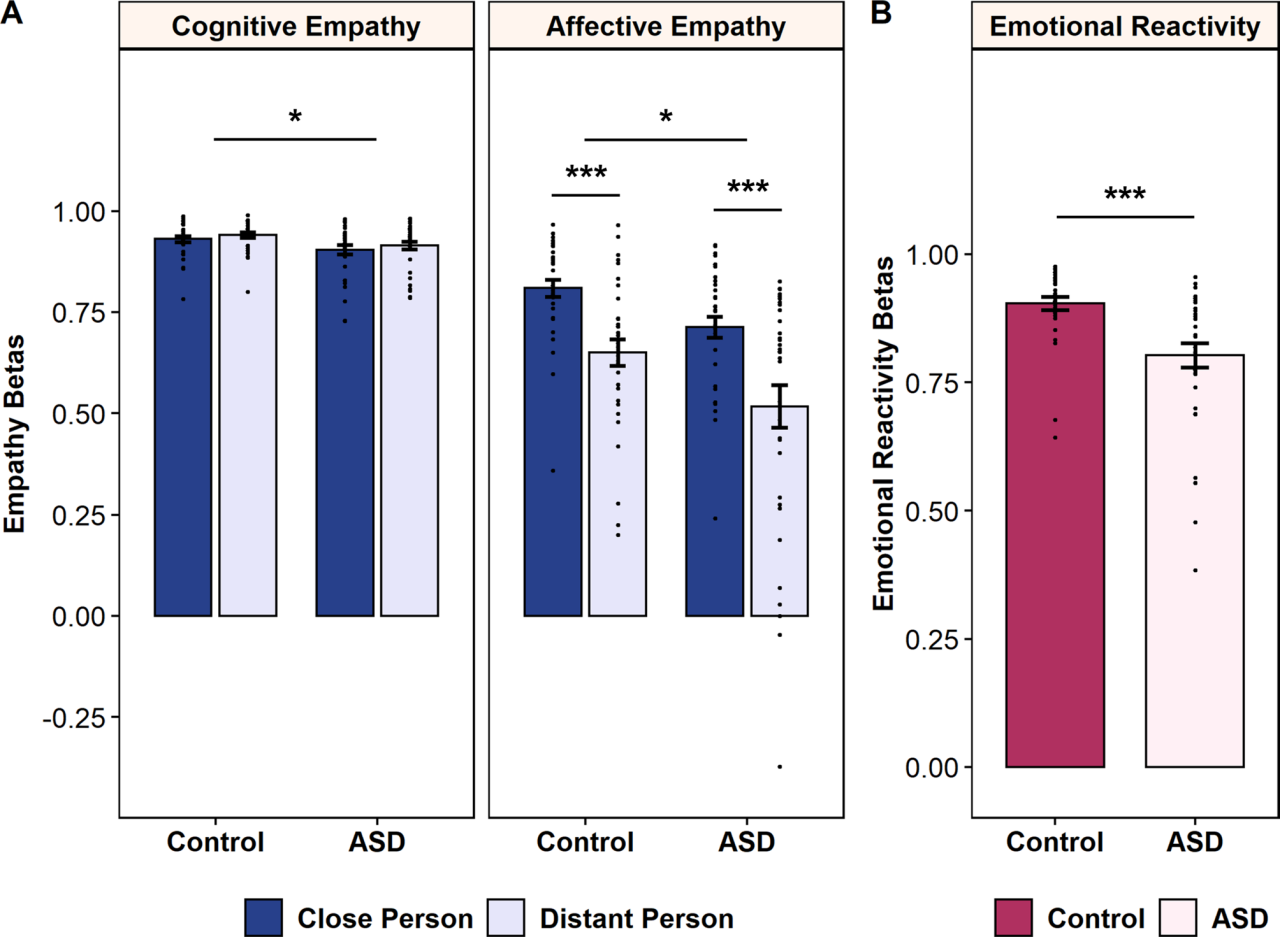
Comparison of cognitive empathy, affective empathy and emotional reactivity between TD controls and autistic individuals. (**A**) shows the cognitive (left) and affective (right) empathy beta weights relating normative (i.e., acquired from an independent sample) self-ratings in emotional situations with imagining how another person (i.e., socially close: dark blue, socially distant: light blue) would feel experiencing the same situation and how the participants feel when imagining another person in this situation, respectively. The bar plot in (**B**) shows the difference in emotional reactivity (i.e., relating normative self-ratings to participant’s self-rating) between TD controls (red) and autistic individuals (light pink). Error bars indicate the mean standard error. Jitter represents the single datapoints per participant. *ASD* autism spectrum disorder, *TD* typically developed, *p < .05, ***p < .001.

The mixed-effects ANOVA on empathic responses revealed a significant main effect of group (F(1,65) = 10.32, p = 0.002, _p_η^2^ = 0.14). The effect of the interaction between group and the component of empathy as well as group and social distance were not significant (group-by-empathy type: F(1,65) = 3.72, p = 0.058, _p_η^2^ = 0.05; group-by-social distance: F(1,65) = 0.52, p = 0.472; group-by-empathy type-by-social distance: F(1,65) = 0.51, p = 0.478). Empathic response betas in autistic persons were significantly smaller compared to TD controls. Hypotheses driven Mann–Whitney U tests revealed that autistic individuals relative to TD controls exhibited significantly lower cognitive (U = 390, p_1-tailed_ = 0.032, η^2^ = 0.07) and affective (U = 375, p_1-tailed_ = 0.020, η^2^ = 0.08) empathic responses as represented by lower betas (see Fig. 1A). Although the ASD group reported significantly greater difficulty in imagining others in emotional situations (close: U = 365.5, p = 0.003, η^2^ = 0.14, distant: U = 406, p = 0.059, η^2^ = 0.05), including these potential confounders as covariates to the between-subjects analysis did not eliminate the group difference in empathic responses (F(1,62) = 5.92, p = 0.018, _p_η^2^ = 0.09). The ability to imagine strangers in emotional scenarios was positively associated with empathic responses (F(1,62) = 7.29, p = 0.009, _p_η^2^ = 0.10). However, within-group analyses revealed no significant associations between the level of empathic responses and the ability to imagine socially close (ASD: *rho*(33) = -0.04, *p* = 1.000; TDC: *rho*(33) = 0.26, *p* = 0.288) or socially distant (ASD: *rho*(33) = 0.24, *p* = 0.280; TDC: *rho*(33) = 0.39, *p* = 0.064) individuals after multiple-comparison correction.

Independently of ASD status, cognitive empathy was significantly higher than affective empathy (main effect empathy type: F(1,65) = 119.55, p < 0.001, _p_η^2^ = 0.65). Furthermore, a main effect of social distance (F(1,65) = 44.88, p < 0.001, _p_η^2^ = 0.41) and a significant interaction effect of social distance-by-empathy type (F(1,65) = 49.02, p < 0.001, _p_η^2^ = 0.43) occurred. Regarding the different types of empathy, post-hoc Wilcoxon signed-rank tests revealed that affective (z = −6.02, p < 0.001, *r* = 0.74), but not cognitive (z = −2.06, p = 0.08, *r* = 0.25) empathic response betas were higher towards socially close than distant persons (see Fig. 1A).

Additional analyses, using the leave-one-out method to tailor TDC and ASD-referenced group means based on participant self-ratings instead of normative means, supported the robustness of the reported findings, particularly for affective empathy (see supplementary information and Figure S1, S2). Standardized regression coefficients indicated the consistency or strength of the association between the expected emotional response of the target person and participants' empathic response. Unstandardized regression coefficients (B values) help understand the slope or magnitude of empathic responses. Mixed ANOVAs with B values showed similar patterns to the analyses using betas, with less pronounced group differences in empathic responses, especially for ASD-referenced B values, where no significant difference between autistic individuals and TD controls was found (see supplementary information, Figure S3–S5).

### Emotional reactivity in autistic individuals and typically developed controls

The hypothesis driven Mann–Whitney U test confirmed that autistic individuals showed on average significantly smaller standardized regression coefficients for emotional reactivity than TD controls (U = 236, p < 0.001, see Fig. 1B). Perceived mental imagery ability during the self-condition was not associated with emotional reactivity within either group (ASD: *rho*(33) = −0.05, *p* = 0.784; TDC: *rho*(33) = 0.15, *p* = 0.149). The finding of reduced emotional reactivity is further supported by supplementary analyses of both referenced betas and B values for TDC and ASD (see supplementary information, Figure S1–S5).

### Effect of ASD diagnosis on empathy mediated by emotional reactivity

**Fig. 2:**
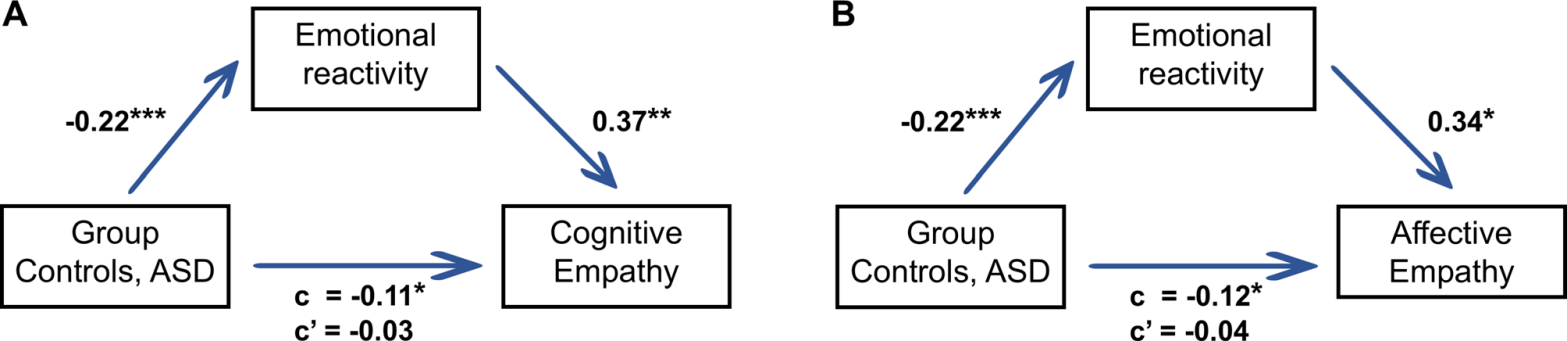
Mediation analyses of group on (**A**) cognitive empathy and (**B**) affective empathy with the mediator emotional reactivity. Empathy and emotional reactivity betas were Box-Cox transformed prior to the analyses to increase linearity. *ASD* autism spectrum disorder, *c* total effect, *c’* direct effect, *p < .05, **p < .01, ***p < .001.

To determine whether the group difference in empathy between ASD and TDC groups is mediated by differences in emotional reactivity, two simplified mediation analyses were conducted separately for cognitive and affective empathy. In each analysis, group was the predictor variable, emotional reactivity was the mediator, and cognitive or affective empathy served as the outcome variable (see Fig. 2). For both models, group significantly predicted empathic responses: cognitive empathy (total effect: c = −0.11, p = 0.017) and affective empathy (total effect: c = −0.12, p = 0.014). When emotional reactivity was included as a mediator in the regression model, ASD status significantly predicted emotional reactivity (a = -0.22, p < 0.001). In turn, emotional reactivity significantly predicted both cognitive empathy (b = 0.37, p = 0.002) and affective empathy (b = 0.34, p = 0.010), while accounting for the group factor (direct relationship without group: b_nogroup_ = 0.40, p < 0.001 and b_nogroup_ = 0.39, p < 0.001, respectively). The direct effects of group on cognitive empathy (c’ = −0.03, p = 0.610) and affective empathy (c’ = −0.04, p = 0.440) were not significant. However, the bootstrapped test of joint indirect effects of group on each component of empathy via emotional reactivity showed significant effects for cognitive empathy (*ab* = −0.08, 95% CI [−0.153, −0.028]) and affective empathy (*ab* = −0.08, 95% CI [−0.139, −0.021]). Therefore, the findings indicate that emotional reactivity mediates the relationship between group and both cognitive and affective empathy, as evidenced by the significant indirect effects and the non-significant direct effects after accounting for the mediator. Notably, there was no significant interaction effect between group and emotional reactivity on empathic responses (cognitive empathy: *F*(1,63) = 1.03, p = 0.313, affective empathy: *F*(1,63) = 3.85, p = 0.054).

## Discussion

The aim of this study was to enhance the understanding of empathy deficits in autistic individuals compared to typically developed controls. To this end, we used the Textual Empathy Test (TET), which is based on textual descriptions of emotional scenarios involving individuals at varying social distances. The TET was designed to capture empathic responses to a broad range of positive and negative emotions while minimizing or accounting for confounding factors such as emotion recognition, emotion matching abilities and emotional reactivity.

In line with our first hypothesis, autistic individuals showed significantly lower levels of both cognitive and affective empathy compared to age-matched typically developed controls in our study, even when empathy was assessed independently of potential perceptual deficits, such as interpreting nonverbal emotional cues from faces, gestures, and voices. Our findings align with a recent meta-analysis^7^ indicating impaired cognitive and affective empathy skills in autistic individuals. However, contrary to previous studies^15,34^, the group differences in empathic responses appear independent of the type of empathy or the social distance to the target person. As expected in our second hypothesis, lower cognitive and affective empathy in autistic individuals were accompanied by aberrant (i.e., lower) emotional reactivity. Further analyses revealed that this aberrant emotional reactivity mediated the effect of ASD status on cognitive and affective empathy. Given the crucial role of empathic skills in social functioning^2^, deficits in empathy may contribute to the communication and social interaction challenges that are core symptoms of ASD. For those affected, this can result in difficulties engaging in conversations or establishing and maintaining social relationships. However, impaired emotional and empathic reactivity does not necessarily lead to reduced prosocial behavior. In autistic individuals, prosocial behaviors guided by cognitive principles, such as fair resource allocation regardless of social distance^39^ and moral decision-making^40^, tend to be more resilient to emotional deficits.

Building on Bird and Viding’s framework^4^, our findings of lower emotional reactivity as well as reduced cognitive and affective empathy in autistic individuals may be linked to an altered understanding of social situations, influenced by atypical social scripts and a distinct representation of affective states. Accordingly, our study indicates a fundamental dysfunction in empathic processes (mediated by aberrant emotional reactivity), rather than a selective impairment in either cognitive or affective empathy. Previous findings on affective empathy deficits in ASD have been mixed. Some studies suggest that affective empathy may be primarily impaired with distant or unfamiliar people, while remaining intact for socially close individuals^15,34^. The similarity hypothesis provides a possible explanation for this observation, positing that socially close individuals tend to be more similar to those with ASD and share comparable social scripts, thereby facilitating empathic processes and social bonding^41^. Relatives of autistic individuals are also likely to exhibit higher levels of ASD-like traits, making them more akin and understandable to autistic individuals compared to strangers^42,43^. Moreover, increased social attention and familiarity with emotional responses of socially close individuals may facilitate empathic responses, whereas relating to strangers could be more challenging. Therefore, it has been argued that autistic individuals may not necessarily experience general empathic deficits but instead demonstrate aberrant empathic behavior depending on the characteristics and social scripts of the target person^44^.

Consistent with previous literature^45,46^, both autistic individuals and TD controls showed significantly higher affective empathic responses for socially close compared to distant persons in the present study. However, we found no evidence supporting a preservation of affective empathy towards socially close targets in autistic individuals compared to TD control subjects. This inconsistency might be explained by the operationalization of affective empathy in our study. We calculated empathic responses using normative mean ratings from an independent sample of TD individuals, likely possessing different social scripts than those imagined by autistic individuals for socially close individuals. This discrepancy could contribute to the lower affective empathy scores observed in ASD. Nevertheless, follow-up analyses presented in the supplementary information, which considered ASD-related social scripts and altered affective responsiveness by using tailored leave-one-out means of the ASD group as predictors for cognitive and affective ratings, also revealed no moderating effect of social distance on group differences in cognitive and affective empathy. Beyond the notion of a selective preservation of affective empathy, some studies even propose an elevated affective empathy (or increased personal distress) in autistic individuals using task-based as well as questionnaire-based self-report and physiological measures (e.g.,^11,17,47^). Our findings provide no support of these findings on a behavioral level, whether examining the consistency of the association between the emotional states of autistic individuals and their target persons (using standardized regression coefficients) or focusing on the slope of this association (using unstandardized regression coefficients, see supplementary information), which represents relative empathic hypo- or hyperreactivity.

In addition to deviations in emotional reactivity and social scripts, factors such as alexithymia (i.e., the inability/difficulty to recognize, express or describe one’s own feelings) and depression may moderate cognitive and affective empathic deficits in autistic individuals^4^. These conditions also influence both physiological and self-reported emotional reactivity^48,49^. Given the relatively high prevalence of alexithymia and depression in autistic individuals (see for meta-analyses:^50,51^), our study could have benefited from assessing these factors to gain a better understanding of additional mediating effects. Consequently, our study design makes it challenging to disentangle how alexithymia and depressive symptoms might have influenced the observed differences in behavioral responses (i.e., subjective valence ratings). Furthermore, the inclusion of physiological measures such as skin conductance and heart rate could have helped to determine whether autistic individuals face challenges solely in subjectively rating their own and others’ feelings or whether these challenges are accompanied by lower physiological arousal. Reduced physiological arousal would provide a more objective measure of diminished affective empathy, thereby strengthening conclusions drawn from behavioral responses alone. Given the text-based nature of the TET, our findings rely on the participants’ ability to engage in mental imagery. However, our study lacked a true non-social imagery control condition, which would have helped account for differences in mental imagery that might explain variations in empathic responses. In fact, autistic participants reported greater difficulty in imagining themselves and others in emotional situations compared to the TD controls. Despite this, perceived difficulty in mental imagery was not correlated with empathic responses and did not account for the observed group differences. Nevertheless, future research should incorporate more objective and detailed measures of mental imagery skills to better control for any potentially confounding effects. Additionally, expanding the TET to assess other facets of empathy, such as compassionate empathy—encompassing both the desire and ability to help—could provide a more comprehensive understanding of empathic abilities in autistic individuals. Lastly, the TET falls short of being a real-life paradigm, creating an artificial setting that may not effectively trigger physiological reactions. However, unlike picture- or video-based tasks or real-life paradigms, as previously recommended for more accurate assessments of affective empathy^6,52^, the TET offers better control over confounding perceptual deficits (e.g., emotion recognition). To achieve a better balance, future studies could explore more naturalistic text-based paradigms. For instance, embedding emotional scenarios within text messages or social media formats could simulate interactions with both socially close and distant individuals.

To advance our understanding of the mechanisms underlying empathic processing in ASD, future studies should explore both peripheral-physiological markers and potential neural mechanisms. For this purpose, the TET could be a promising tool to be applied in an fMRI environment (see^24^). If future studies validate our current findings of a mediated decrease in empathic response due to aberrant emotional reactivity, likely stemming from atypical social scripts, and unveil the underlying neural substrates, this could hold significant therapeutic implications. For example, socio-cognitive training aimed at incorporating neurotypical social scripts may enhance empathic responses and, consequently, improve social functioning (e.g.,^53^). Furthermore, a more profound understanding of neural substrates linked to ASD-related challenges could guide the identification of promising therapeutic targets using neural modulation techniques (e.g., transcranial magnetic stimulation [TMS], transcranial direct current stimulation [tDCS], or neurofeedback). In fact, initial neuromodulation studies employing TMS and tDCS, primarily targeting the dorsolateral PFC, show some promise in alleviating ASD symptoms and improving social functioning^54,55^.

In conclusion, the present findings suggest that lower cognitive and affective empathic responses in autistic individuals could be mediated by differences in emotional reactivity. While a greater dissonance exists between the empathic response and the feelings of socially distant individuals compared to close ones, there is no indication of a selective preservation of affective empathy in autistic individuals toward socially close individuals. Additionally, there were no quantitative disparities in ASD-related deficits in cognitive and affective empathy. Ultimately, gaining a deeper understanding of the underlying mechanisms of the suggested ASD-related empathy deficits could contribute to the development of more tailored therapeutic interventions aimed at improving social functioning.

## Methods

### Participants

In total, 69 individuals participated in the study. Two participants were excluded due to data loss or insufficient German language skills, resulting in 34 autistic individuals and 33 typically developed controls. The ASD group was recruited via the out-patient consultation service specialized for autistic adults of the Department of Psychiatry and Psychotherapy at the University Hospital Tübingen.

For inclusion, participants had either been diagnosed with high-functioning early childhood autism (F84.0) or Asperger-Syndrome (F84.5) according to the ICD-10 criteria by fully trained clinicians using extensive clinical examinations. These examinations consisted of a comprehensive anamnesis, an evaluation of interpersonal behavior and a battery of different questionnaires completed by the autistic participants including the autism-spectrum quotient (AQ)^56^, and the multiple-choice vocabulary intelligence test (MWT-B)^57^. Furthermore, questionnaires on the participant’s (social) behavior in the first decade of their life were completed by at least one relative who witnessed this period. These questionnaires included the social responsiveness scale (SRS)^58^, the social communication questionnaire (SCQ/FSK)^59^ and the Marburg rating scale for Asperger’s syndrome (MBAS)^60^ and complemented the clinical diagnosis.

### Procedure

The study was carried out at the Department of Psychiatry and Psychotherapy of the University of Tübingen. During diagnostics, autistic individuals gave consent to be informed about clinical studies and were subsequently contacted via email. For the TDC group, non-autistic, typically developed participants were matched to the ASD group with respect to age, education, and IQ (see Table 1). They were recruited via flyers distributed in Tübingen (Germany). Prior participation, all participants were informed about the study design, its voluntary nature, as well as data protection and gave informed consent. The current version of the computer based Textual Empathy Test (TET) lasted approximately 20 min. After completion of the TET, we asked all participants to rate the type of person and the social distance towards him/her, to ensure that autistic individuals and healthy controls chose comparable socially close and socially distant target persons when imaging the emotional scenarios (see supplementary information). Additionally, all participants reported their perceived difficulty (categorized as difficult, medium, or easy) in imagining themselves, the respective socially close person and socially distant person in the emotional situations. Beyond the TET, participants completed a battery of written questionnaires measuring socio-demographic data such as age and education, verbal intelligence (MWT-B)^57^, and the degree of ASD symptoms (AQ)^56^ to ensure that the groups are comparable with regards to socio-demographic characteristics and that the TDC group presents a significantly lower autism-spectrum quotient (AQ). For the ASD group, the AQ and verbal intelligence had already been determined prior to the study during the diagnostic session. In total, the appointment lasted approximately 1.5 h and was financially compensated with 10 Euro/hour. The study was approved by the Ethics committee of the Medical Faculty of the University Tübingen and followed the ethical code of the World Medical Association (Declaration of Helsinki).

### Development of the Textual Empathy Test (TET)

The textual stimuli of emotional everyday situations of the former Tübinger Empathy Test^24,36^ were revised to be compatible for both sexes and a wider age range. Furthermore, in addition to the existing emotional categories (positive: joy, sexual pleasure, gratefulness; negative: anger, disgust, fear), they were extended by short descriptions of everyday situations evoking hope, pride, relief, sadness, shame, and envy to capture empathic abilities with respect to a broad range of everyday emotional situations. Information on the validation and an overview of the final stimuli set is provided in the supplementary information.

**Fig. 3:**
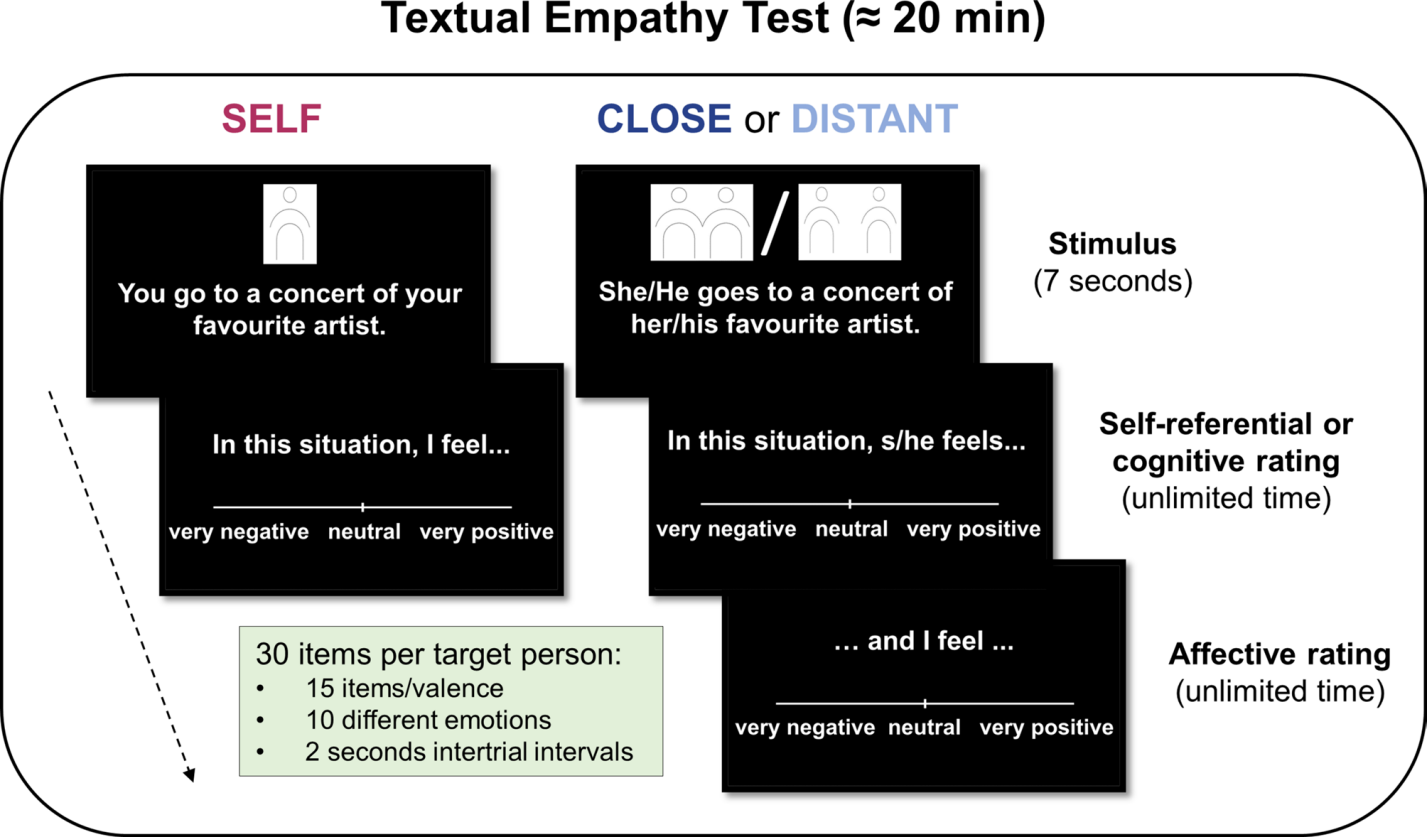
Illustration of the Textual Empathy Test (TET). The TET consists of three blocks (i.e., self, close and distant person as target person, respectively) with 30 textual descriptions of emotional situations each. Described emotional situations were balanced for positive and negative emotions. Each stimulus was shown for 7 s. After stimulus presentation, participants were asked to rate on a visual analogue scale (VAS) from very negative to very positive how the target person would feel in such a situation (i.e., cognitive rating). For the close and distant target person, participants were then asked with another VAS how the participant feels, when this happens to the target person (i.e., affective rating). Each trial was followed by a fixation cross lasting 2 s.

The final selection for the Textual Empathy Test (TET) encompassed 30 short descriptions of emotional situations (i.e., 3 items per emotional category) tapping into 5 positive (joy, hope, sexual pleasure, gratefulness, pride) and 5 negative emotions (shame, anger, disgust, sadness, fear). Examples include: “She/he fought hard for his goals and achieved them.” and “She/he hears about the death of a person, she/he liked very much.” Items for relief and envy did not pass inclusion criteria, which resulted in the drop of these emotional categories. The 30 described emotional situations were repeatedly presented in three blocks, each relating to the self and to a different target person with varying degrees of social distance (partner/relative/friend, stranger). Participants were asked to rate how they would feel in that respective situation (self), how the other person would feel (cognitive rating) and which feeling they experience themselves when imagining the other person in that situation (affective rating; see Fig. 3 for illustration of the TET). The sequence of items within the blocks was randomized. Each item was shown for 7 s followed by a fixation cross (1 s) and visual analogue scale (VAS) to assess the valence ratings (i.e., one VAS for self-condition; two VAS for each other-perspective, separated by a 1 s fixation cross). Ratings from -100 (very negative) to +100 (very positive) and response times were recorded through computer mouse movements and button presses. There was no time limit for the response. An answer was mandatory to continue with the next trial. Each trial was followed by an 2 s fixation cross.

### Data analysis

Data analysis was run using IBM SPSS Statistics 25 (IBM, New York). For all analyses, two tailed testing with an α–level of 0.05 was applied, if not otherwise specified.

#### Demographic and neuropsychological information

To demonstrate comparability of the ASD and TDC group in terms of demographic and neuropsychological factors including age, sex, educational level and verbal intelligence, analyses for group differences were carried out. AQ scores were analyzed to compare autistic traits in both groups with the expectation that these are significantly higher in the ASD group. Mann–Whitney U tests were used for group analyses regarding age, verbal intelligence, and ASD traits (i.e., AQ scores), as data was not normally distributed. Chi-square tests were run for sex (male/female) and educational level (i.e., achieved academic degree—yes/no) with group as the between-subject factor.

#### TET: computation of empathic responses and emotional reactivity

Cognitive and affective empathy were computed per participant by separate regression analyses using (normative) mean ratings of the self-condition collected in an independent sample (n = 82) as the predictor for (1) the self-ratings of this study’s participants (i.e., emotional reactivity; very negative to very positive), (2) the cognitive ratings for the target person’s emotional state in the same situation (i.e., cognitive empathy; very negative to very positive) and (3) the affective ratings of the emotional state they themselves are in when imagining the target person in this situation (i.e., affective empathy; very negative to very positive). Cognitive and affective empathy were thus operationalized by the resulting standardized regression coefficients, which were generated separately for close and distant condition (see for more detail: Kimmig et al.^36^). For one autistic individual the standardized regression weight for affective empathy towards a stranger was set to 0, as each item was rated as neutral when asked how the participant would feel when imagining the stranger in this situation.

##### Independent normative sample

Participants of the independent sample were recruited via convenience sampling using social media, most notably the student Facebook group of the Hochschule Fresenius in Frankfurt (Germany). The study was targeted at (mentally) healthy individuals only. The sex of participants was equally distributed, and their age ranged from 18 to 36 years (*m* = 24.3, *sd* =  ± 4.0). Most participants were university students (63.4%) or currently employed (29.3%).

The TET was employed using the SoSci Survey software (SoSci Survey GmbH).

#### Group differences in empathic responses and emotional reactivity

To examine empathic responses, the standardized regression coefficients (i.e., betas) were entered as dependent variables into a mixed-effects ANOVA. The mixed-effects ANOVA consisted of the within-subject factors empathy component (cognitive, affective) and personal distance (close, distant) and the between-subjects factor group (ASD, TDC). In case of non-normally distributed data, Mann–Whitney U or Wilcoxon tests instead of parametric t-tests were used for post-hoc analyses. Bonferroni correction was applied in these analyses to correct for multiple testing. Standardized regression coefficients for emotional reactivity were analyzed by a Mann–Whitney U test.

To test the robustness of our findings, we ran similar analyses using as reference for the regression coefficients the participant-tailored leave-one out TDC and ASD group means instead of the normative means of an independent sample (see supplementary information, Figure S1, S2). Moreover, the supplementary information also includes complementary analyses of the unstandardized regression coefficients (see supplementary information, Figure S3–S5).

#### Mediation analyses of ASD diagnosis and emotional reactivity on empathy

Two simple post-hoc mediation analyses (independent variable: group, mediator: Box-Cox transformed emotional reactivity betas, dependent variable: Box-Cox transformed cognitive or affective empathy betas, respectively; see Fig. 2) were run to determine whether an effect of group on empathic responses could be mediated by emotional reactivity. Box-Cox transforms were used to increase linearity of the data’s relationships. The analyses were performed using Hayes Process v4.2 (http://www.processmacro.org/download.html) employing model 4 with 5000 bootstrap iterations in SPSS.

## Supplementary Information

Supplementary Information.

## Data Availability

The data that support the findings of this study are available from the corresponding author upon reasonable request.
