# Supplementary material for: Impairment of affective and cognitive empathy in high functioning autism is mediated by alterations in emotional reactivity
Source: Sci Rep. 2024 Sep 17;14:21662. doi: 10.1038/s41598-024-71825-1 (PMC11408716; doi:10.1038/s41598-024-71825-1)
Supplement: Supplementary file 1 — Supplementary Information. [file 41598_2024_71825_MOESM1_ESM.pdf]

Supplementary Information to 'Impairment of affective and cognitive empathy in high functioning autism is mediated by alterations in emotional reactivity'

Kimmig A-C.S.*, Burger L., Schall M., Derntl B., & Wildgruber D.

1. Supplementary Methods

Development and Validation of the Textual Empathy Test

A total of 60 short descriptions of everyday situations including positive (i.e., joy, hope, sexual pleasure, gratefulness, pride, relief; 5 items each) and negative emotions (i.e., shame, anger, disgust, sadness, fear, envy, 5 items each) were adapted from previous studies[1-3] or newly added to the existing stimuli set. Subsequently these stimuli were rated by 22 German speaking participants (13 females, 9 males) recruited via convenience sampling using paper pencil questionnaires. The participants' age ranged from 18 to 72 years (*m*=37.7, *sd*=±17.8) and the majority was either currently employed or were students. The participants were instructed to imagine themselves in the respective situations. Stimuli were presented in a randomized order. After reading the emotional situation carefully, they had to rate the level of valence of their emotional response on a seven-point emoji-based visual analogue scale from very negative to very positive (A=very positive to G=very negative). Moreover, the participants had to choose one of the 12 emotional categories (joy, hope, sexual arousal, gratefulness, pride, relief, shame, anger, disgust, sadness, fear, envy) most accurately reflecting the emotion evoked by the described everyday situation. Only stimuli which were correctly identified (i.e., emotional category) by at least 70% of participants and had congruent valence ratings qualified for the inclusion to the TET. Not enough items of relief and envy did pass this requirement; therefore, these emotions were not included in the final selection of stimuli.

Table S1 provides an overview of all emotional scenarios in German and its English translation of the close and distant target person conditions. For the self-condition, the pronouns and verb form should be adjusted accordingly.

Example:

Stimulus for socially close or distant target persons (marked with respective symbols):

> Sie/er geht auf das Konzert ihres/seines Lieblingsmusikers.
> *She/he is going to the concert of her/his favorite musician.*

Stimulus for self-condition:

> Ich gehe auf das Konzert meines Lieblingsmusikers.
> *I am going to the concert of my favorite musician.*

The self-referential, cognitive and affective ratings for these emotional scenarios were assessed with visual analogue scales from very negative to very positive by the following statements:

- Self-referential and cognitive ratings:
  In der beschriebenen Situation FÜHLT sich DIE PERSON…
  In the described situation, THIS PERSON feels…
- Affective ratings:
  Dabei FÜHLE ICH mich…
  Now I FEEL…

Table S1. Overview of emotional scenarios included in the TET in German and English

**TET Stimuli for close and distant target persons** **for self-condition: change to I (ich), me (mich), my (mein), myself (mir/mich) or mine (meines/mir) and adjust verb form accordingly**
**Positive Emotions**
**Joy**
(1) Sie/er erhält beim Preisausschreiben den Hauptgewinn.<br>*She/he receives the main prize at the sweepstake.*<br>(2) Sie/er geht auf das Konzert ihres/seines Lieblingsmusikers.<br>*She/he is going to the concert of her/his favorite musician.*<br>(3) Sie/er sieht einen guten Freund nach langer Zeit wieder.<br>*She/he is seeing a good friend again after a long time.*
**Hope**
(1) Sie/er ist schwer krank, es wird eine neue Behandlungsmethode ausprobiert.<br>*She/he is seriously ill, a new treatment method is being tested.*<br>(2) Sie/er hat die Möglichkeit, bei einem Preisausschreiben eine Traumreise zu gewinnen.<br>*She/he has the opportunity to win a dream journey in a sweepstake.*<br>(3) Sie/er spielt Lotto. Die ersten 3 Zahlen, die vorgelesen werden, hat sie/er richtig getippt.<br>*She/he plays the lottery. The first three numbers that are read out, she/he has guessed correctly.*
**Sexual Pleasure**
(1) Mit jeder weiteren Berührung wird ihr/sein Körper erregter.<br>*With each additional touch, her/his body becomes more aroused.*<br>(2) Die Liebkosungen lassen sie/ihn vor Erregung beben.<br>*The caresses make her/him tremble with excitement.*<br>(3) Sie/er kann sich vor Lust kaum noch halten.<br>*She/he can hardly contain herself/himself with desire.*
**Gratefulness**
(1) Sie/er liegt krank im Bett, jemand besorgt Medikamente.<br>She/he is sick in bed, someone buys medication.<br>(2) Jemand hilft ihr/ihm bei der Erledigung einer wichtigen Aufgabe.<br>*Someone helps her/him to complete an important task.*<br>(3) Sie/er läuft mit Krücken, jemand hält die Tür auf.<br>*She/he is walking with crutches, someone holds the door open.*
**Pride**
(1) Sie/er schafft es, einen Marathon durchzulaufen.<br>*She/he manages to complete a marathon.*<br>(2) Sie/er hat jemand Schwächeren vor anderen verteidigt.<br>*She/he defended someone weaker from others.*<br>(3) Sie/er hat für ein Ziel hart gekämpft und es erreicht.<br>*She/he fought hard for a goal and achieved it.*

Negative Emotions
**Anger**
(1) Ihr/sein Nachbar hat trotz Aufforderung die Musik nicht leiser gestellt.<br>*Despite being asked, her/his neighbor did not turn down the music.*<br>(2) Ihr/sein Geldbeutel wird auf einer belebten Straße gestohlen.<br>*Her/his wallet is stolen on a busy street.*<br>(3) Ihre/seine Bahn hat Verspätung, sodass sie/er den Anschlusszug verpasst.<br>*Her/his train is delayed, so she/he misses the connecting train.*
**Disgust**
(1) Sie/er muss eine verschmutzte Toilette benutzen.<br>*She/he has to use a dirty toilet.*<br>(2) Neben ihr/ihm im Schwimmbad liegt eine Person mit Fußpilz.<br>*Next to her/him at the swimming pool, a person with athlete's foot is lying.*<br>(3) Sie/er sitzt in einer vollbesetzten Bahn, plötzlich verbreitet sich ein übelriechender Geruch.<br>*She/he is sitting in a crowded train, suddenly a foul-smelling odor spreads.*
**Fear**
(1) Nachts auf dem Heimweg bemerkt sie/er schnelle Schritte hinter sich.<br>*At night on the way home, she/he notices fast footsteps behind her/him.*<br>(2) Sie/er bekommt plötzlich Schwierigkeiten beim Atmen.<br>*She/he suddenly has trouble breathing.*<br>(3) Ihr/sein Flugzeug sackt in der Luft gewaltig ab.<br>*Her/his airplane plummets significantly in the air.*
**Sadness**
(1) Sie/er erfährt vom Tod einer Person, die sie/er sehr gerne gehabt hat.<br>*She/he learns of the death of a person he/she was very fond of.*<br>(2) Ihre/seine Katze muss auf Grund einer Krankheit eingeschläfert werden.<br>*Her/his cat has to be put down due to an illness.*<br>(3) Ihr/ihm zerbricht ein altes Erinnerungsstück.<br>*An old keepsake of hers/his breaks.*
**Shame**
(1) Sie/er bückt sich in der Öffentlichkeit, alle sehen wie ihre/seine Hose am Po reißt.<br>*She/he bends over in public, everyone sees her/his pants rip at the back.*<br>(2) Sie/er wird beim Sex erwischt.<br>*She/he gets caught having sex.*<br>(3) Sie/er verschickt eine intime Nachricht versehentlich an ihren/seinen Chef.<br>*She/he accidentally sends an intimate message to her/his boss.*

2. Supplementary Analyses

TDC- and ASD-referenced Cognitive Empathy, Affective Empathy and Emotional Reactivity using Standardized Regression Coefficients

The 'double empathy problem' proposes that individuals with ASD may not necessarily experience overall deficits in empathy. Instead, they might display atypical empathic behavior[4]. It is anticipated that there would be no deficits in empathy when connecting with others who also have ASD or share similar social scripts. To test whether group differences found in the main analyses are dependent on the reference of the predictor (i.e., independent neurotypically developed sample), we did additional analyses taking the means of self-ratings of the typically developed control (TDC) and ASD group, respectively, as predictors to generate the standardized regression coefficients. Here we used a leave-one-out approach to calculate the group means of each item in the self-condition individually by omitting the respective participant's own self-ratings. The standardized regression coefficients for the empathic responses were then entered into a 2(group: TDC, ASD) by 2(empathy component: cognitive, affective) by 2(personal distance: close, distant) mixed ANOVA model, while the standardized regression coefficients for emotional reactivity were analyzed with a Mann-Whitney U tests due to non-normally distributed data.

TDC-referenced betas. The results of the mixed ANOVA and the Mann-Whitney U test using the control-referenced standardized regression coefficients for empathic responses and emotional reactivity, respectively, reveal similar patterns to the normative standardized regression coefficients reported in the main manuscript (see Figure S1 and compare to Figure 2). Furthermore, autistic individuals had significantly lower empathy-related betas (main effect group: $F(1,65)=9.88$, $p=.003$, $_p\eta^2=.13$), which were confirmed by Bonferroni-corrected Mann Whitney U tests for the cognitive ($U=399$, $p_{1tailed} = .042$, $\eta^2=.06$) as well as the affective component of empathy ($U=378$, $p_{1tailed} = .022$, $\eta^2=.08$). There were no significant interactions of group with empathy component and/or social distance (all $|F|\leq3.68$, all $p\geq.059$). Similarly, emotional reactivity betas were significantly lower in the ASD group compared to the TDC group ($U=252$, $p<.001$, $\eta^2=.23$). These patterns support the findings of the main analysis using standardized regression coefficient based on an independent sample.

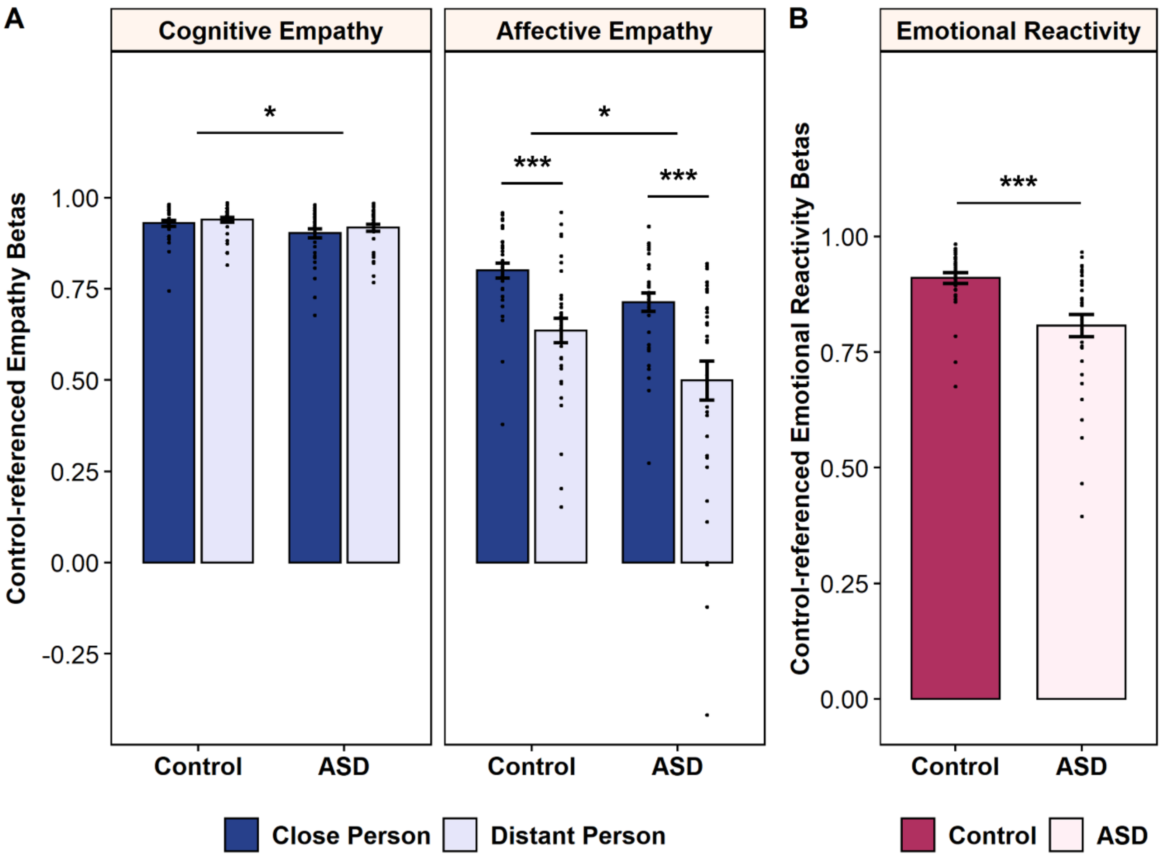

Figure S1. Comparison of TDC-referenced cognitive empathy, affective empathy and emotional reactivity between TD controls and autistic individuals***.*** *A) shows the cognitive (left) and affective (right) empathy beta weights representing the correlation between leave-one-out self-rating means of TD controls and the individual ratings of how another person (socially close in dark blue, socially distant in light blue) would feel in a given emotional situation and how the participants feel when imagining another person in this situation, respectively. The bar plot in B) shows the difference in emotional reactivity (i.e., leave-one-out self-rating means of TD controls to participant's self-rating) between TD controls (red) and autistic individuals (light pink). Error bars indicate the mean standard error, and jitter represents individual data points per participant. ASD – autism spectrum disorder, TD – typically developed control, *p<.05, ***p<.001.*

ASD-referenced betas. Taking the mean ratings of autistic individuals on how they feel if they experienced a certain emotional situation as a predictor for standardized regression coefficients on empathic responses and emotional reactivity did not affect the results considerably. Betas representing empathic responses and emotional reactivity, were significantly lower within the ASD group compared to the TDC group (U=364, p = .013, η^2=.09 and U=290, p<.001, η^2=.18, respectively). Especially, the associations between ASD-related mean self-ratings and individual affective empathic responses as well as emotional reactivity are consistently stronger for TD controls compared to autistic individuals (Bonferroni-corrected Mann-Whitney U tests for cognitive empathy: U=454, p=.36,

affective empathy: U=398, $p_{1tailed}$ = .041, η^2=.06). There were no significant group-related interactions (all |*F*|≤3.32, all *p*≥.073).

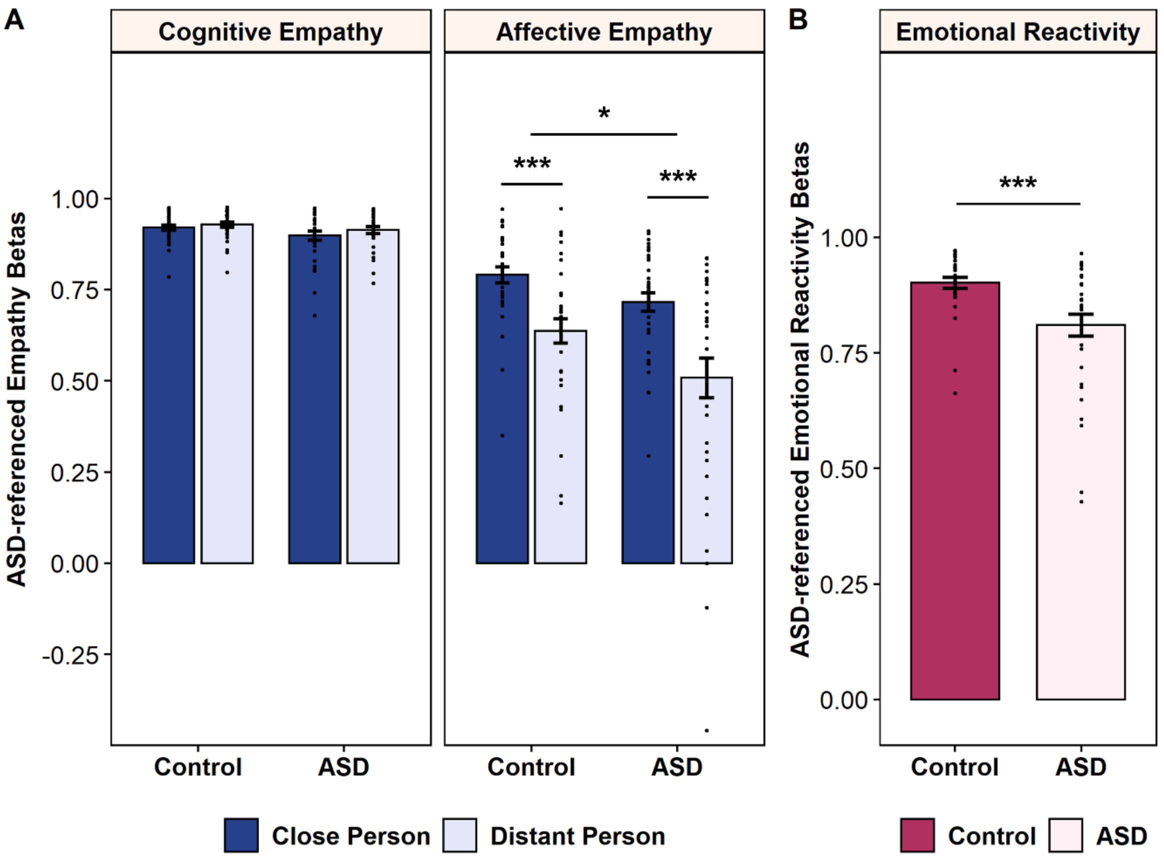

Figure S2. Comparison of ASD-referenced cognitive empathy, affective empathy and emotional reactivity between TD controls and autistic individuals (ASD). *A) shows the cognitive (left) and affective (right) empathy beta weights representing the correlation between leave-one-out self-rating means of autistic individuals and the individual ratings of how another person (socially close in dark blue, socially distant in light blue) would feel in a given emotional situation and how the participants feel when imagining another person in this situation, respectively. The bar plot in B) shows the difference in emotional reactivity (i.e., leave-one-out self-rating means of autistic individuals to participant's self-rating) between TD controls (red) and* autistic individuals *(light pink). Error bars indicate the mean standard error, and jitter represents individual data points per participant. ASD – autism spectrum disorder, TD – typically developed, *p<.05, ***p<.001.*

Unstandardized Regression Coefficients: An Additional Dimension to the Conceptualization of Empathic Responses and Emotional Reactivity

While standardized regression coefficients provide information on the strength/consistency of a linear association between two variables, the unstandardized regression coefficient, the B value, informs about the slope of the relationship. Therefore, complementary analyses (i.e., mixed ANOVA and independent t or Mann-Whitney U tests) with the B values could provide additional information on empathic responses and emotional reactivity such as hypo- (B < 1) and hyperresponsivity (B > 1)

relative to normative, TDC-referenced and ASD-referenced subjective feelings evoked by the respective emotional situations.

Normative Bs. The mixed ANOVA and the independent t-tests with the normative unstandardized regression coefficients (B values) revealed similar patterns for empathic responses and emotional reactivity as previous analyses with the standardized regression coefficients (see Figure S3 and compare with Figures 2, S1-2). Empathic responses ($F(1,65)=3.12$, $p_{1tailed}=.041$, ${}_{p}\eta^2=.05$) and emotional reactivity ($t(65)=3.14$, $p = .003$, $\eta^2=.13$) represented by B values were significantly higher in the TD controls compared to autistic individuals. However, Bonferroni corrected Mann-Whitney U tests did not reveal a statistically significant group difference for cognitive ($U=517$, $p_{1tailed} = .581$) and affective empathy ($U=427$, $p_{1tailed} = .093$) separately. No significant interactions involving group were found (all $|F|\leq0.91$, all $p\geq.344$).

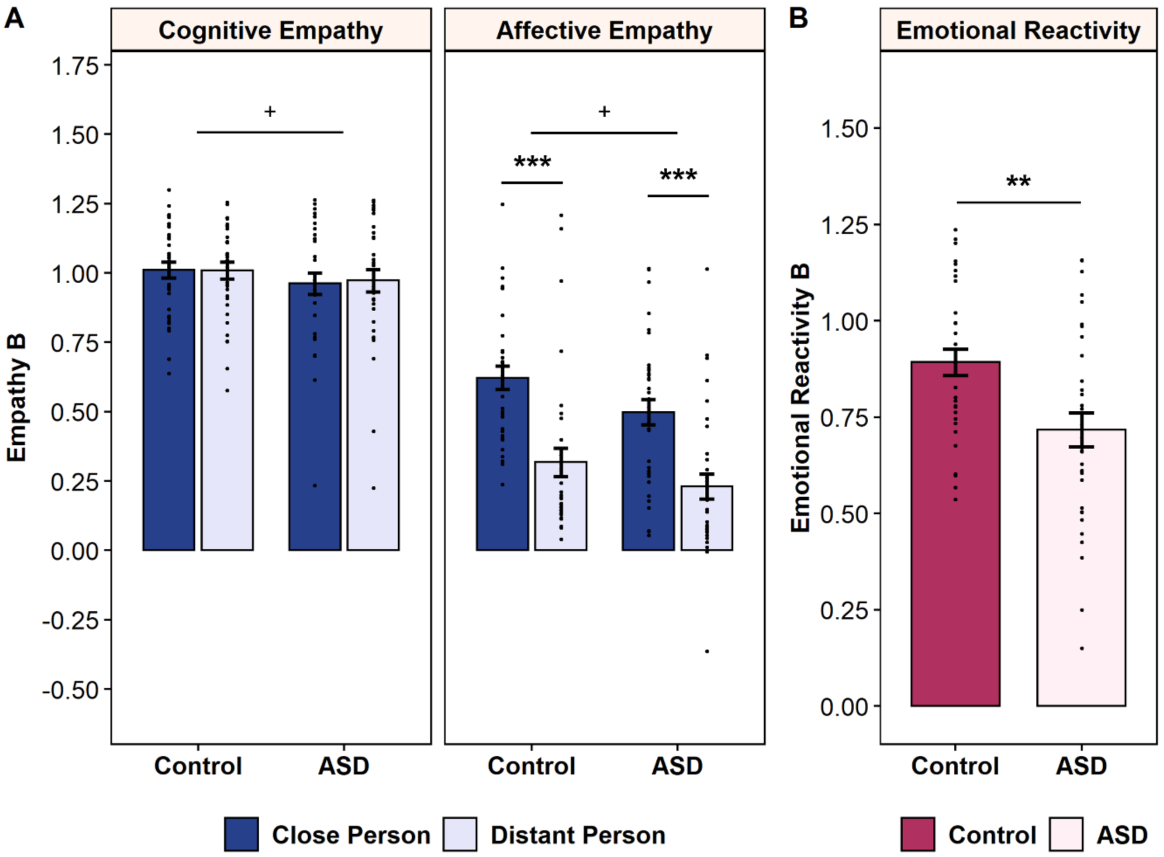

Figure S3. Unstandardized regression coefficients (B) as indicators of cognitive empathy, affective empathy and emotional reactivity between TD controls and autistic individuals (ASD). *A) shows the cognitive (left) and affective (right) empathy B values representing the slope of the correlation between normative (i.e., acquired from an independent sample) self-ratings in emotional situations and the individual ratings of how another person (socially close in dark blue, socially distant in light blue) would feel in a given emotional situation and how the participants feel when imagining another person in this situation, respectively. The bar plot in B) shows the difference in emotional reactivity B values (i.e., relating normative self-ratings to participant's self-rating) between TD controls (red) and autistic*

*individuals (light pink). Error bars indicate the mean standard error, and jitter represents individual data points per participant. ASD – autism spectrum disorder, TD – typically developed, $^{+}p_{maineffect}$<.05, **p<.01, ***p<.001.*

TDC-referenced Bs. Like the normative B values, the TDC-referenced unstandardized regression coefficients were significantly lower in autistic individuals compared to TD controls for both empathic responses and emotional reactivity (see Figure S4, $F(1,65)=3.13$, $p_{1tailed}=.041$, ${}_{p}\eta^2=.05$ and $t(65)=3.15$, $p = .003$, $\eta^2=.13$, respectively). There were no significant interactions of group with empathy component and/or social distance (all $|F|\leq 0.91$, all $p\geq .345$).

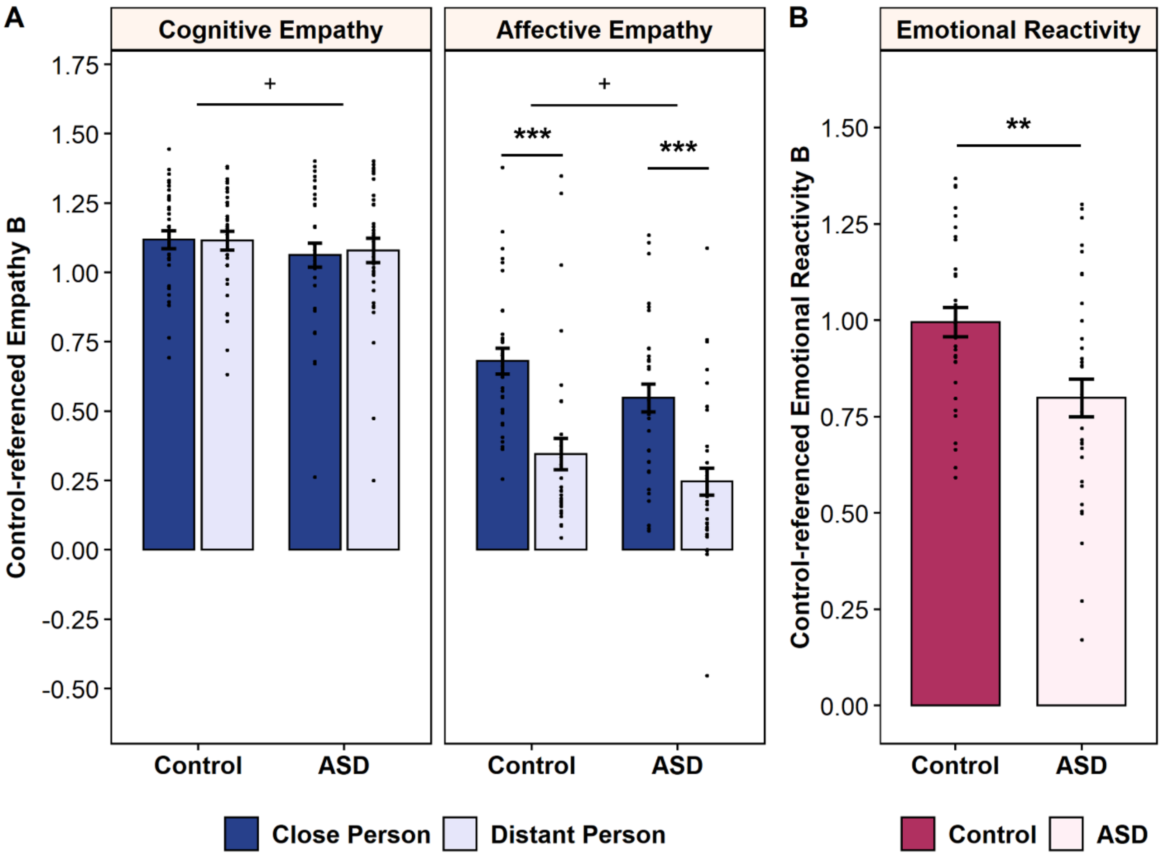

***Figure S4. TDC-referenced unstandardized regression coefficients (B) as indicators of empathy, affective empathy and emotional reactivity between TD controls and autistic individuals (ASD).** A) shows the cognitive (left) and affective (right) empathy B values representing the slope of the correlation between leave-one-out self-rating means of TD controls and the individual ratings of how another person (socially close in dark blue, socially distant in light blue) would feel in a given emotional situation and how the participants feel when imagining another person in this situation, respectively. The bar plot in B) shows the difference in emotional reactivity B values (i.e., leave-one-out self-rating means of TD controls to participant's self-rating) between TD controls (red) and autistic individuals (light pink). Error bars indicate the mean standard error, and jitter represents individual data points per participant. ASD – autism spectrum disorder, TDC – typically developed control, $^{+}p_{maineffect}$<.05, **p<.01, ***p<.001.*

ASD-referenced Bs. While the patterns of the ASD-referenced Bs reflect the patterns also observed for the standardized and unstandardized regression coefficient analyses, there was only a significant group difference for emotional reactivity ($t(65)=2.92$, $p = .005$, $\eta^2=.12$), with TD controls having significantly higher emotional reactivity compared to autistic individuals (see Figure S5). No such group-related difference reached significance for the empathic response B values (main effect group: $F(1,65)=2.42$, $p=.124$). No significant interaction effects with group were detected (all $|F|\leq 0.93$, all $p\geq .339$). Coefficients of higher than 1 for the emotional reactivity indicate that when based on ASD-related standards, TD controls would be classified as emotionally hyperreactive. A similar observation can be made for the cognitive empathy B values, however, here both groups show B vales higher than 1.

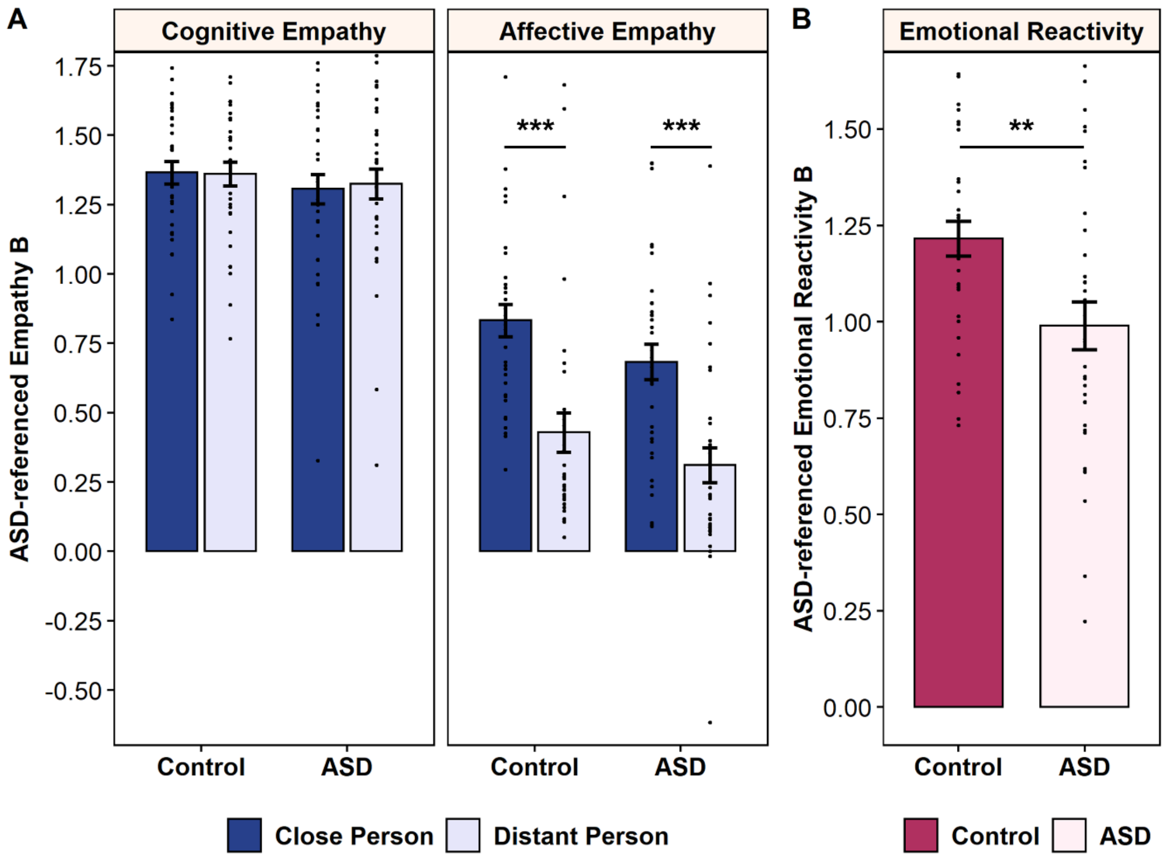

Figure S5. ASD-referenced unstandardized regression coefficients (B) as indicators of cognitive empathy, affective empathy and emotional reactivity between TD controls and autistic individuals (ASD). *A) shows the cognitive (left) and affective (right) empathy B values representing the slope of the correlation between leave-one-out self-rating means of autistic individuals and the individual ratings of how another person (socially close in dark blue, socially distant in light blue) would feel in a given emotional situation and how the participants feel when imagining another person in this situation, respectively. The bar plot in B) shows the difference in emotional reactivity B values (i.e., leave-one-out self-rating means of autistic individuals to participant's self-rating) between TD controls (red) and autistic individuals (light pink). Error bars indicate the mean standard error, and jitter represents*

*individual data points per participant. ASD – autism spectrum disorder, TD – typically developed, **p<.01, ***p<.001.*

Comparability of imagined target persons among autistic individuals and typically developed controls

To inform interpretation of analyses on empathic responses, chi-square and exact Fisher's tests were used to examine the comparability of target persons imagined by the two different groups with respect to type of target person (close: relative/partner, social contact; distant: nobody, stranger from shops/street) and personal distance (close, medium, far).

There was no significant group difference in the type of target persons imagined for the close ($X^2(2)=4.46$, p=.11) nor for the distant person (p=1.00, Fisher's exact test). For the close person, most participants imagined a social contact with familial relationship (47%), followed by imagining a social contact without familial relationship (27%). The remaining participants (26%) imagined multiple persons including both former groups. For the distant person, the majority thought off a stranger they encountered on the street or in a shop (58%), the remaining participants referred to nobody in particular (41%) or imagined multiple persons from both former groups (1%).

Regarding the level of social distance towards the imagined target person, exact Fisher test did also not reveal a significant difference between the ASD and the TDC group for the close (p=1.00, Fisher's exact test) nor the distant person (p=.43, Fisher's exact test). Across both groups, the relationship for the close person was rated as close (91%) or medium (9%), whereas the relation to the distant person was rated as distant (86%) or medium (11%), respectively.

References

1 Kimmig, A.-C. S. *et al.* Lower affective empathy in oral contraceptive users: a cross-sectional fMRI study. *Cereb Cortex*, doi:10.1093/cercor/bhac345 (2022).

2 Kimmig, A.-C. S., Wildgruber, D., Wendel, S.-M. U., Sundström-Poromaa, I. & Derntl, B. Friend vs. Foe: Cognitive and Affective Empathy in Women With Different Hormonal States. *Front Neurosci* **15**, 608768-608768, doi:10.3389/fnins.2021.608768 (2021).

3 Derntl, B. *et al.* Generalized deficit in all core components of empathy in schizophrenia. *Schizophrenia Research* **108**, 197-206, doi:10.1016/j.schres.2008.11.009 (2009).

4 Mitchell, P., Sheppard, E. & Cassidy, S. Autism and the double empathy problem: Implications for development and mental health. *British Journal of Developmental Psychology* **39**, 1-18, doi:10.1111/bjdp.12350 (2021).
